# Health and disease phenotyping in old age using a cluster network analysis

**DOI:** 10.1038/s41598-017-15753-3

**Published:** 2017-11-15

**Authors:** Jesus Felix Valenzuela, Christopher Monterola, Victor Joo Chuan Tong, Tze Pin Ng, Anis Larbi

**Affiliations:** 1 Institute of High Performance Computing, Computing Science Department, 1 Fusionopolis Way, #16-16 Connexis North, 138632 Singapore, Singapore; 2 School of Innovation, Technology and Entrepreneurship, Asian Institute of Management, 123 Paseo de Roxas, Manila, Philippines; 3 Institute of High Performance Computing, Social and Cognitive Computing Department, 1 Fusionopolis Way, #16-16 Connexis North, 138632 Singapore, Singapore; 4 Yong Loo Lin School of Medicine, National University of Singapore, Department of Psychological Medicine, 1E Kent Ridge Road, NUHS Tower Block, Level 9, 119228 Singapore, Singapore; 5 Singapore Immunology Network, 8A Biomedical Grove, Immunos Level 4, 138648 Singapore, Singapore; 6 Department of Medicine, University of Sherbrooke, Quebec, Canada; 7 Yong Loo Lin School of Medicine, National University of Singapore, Department of Microbiology and Immunology, Singapore, Singapore; 8 School of Biological Sciences, Nanyang Technological University (NTU), Singapore, Singapore; 9 Department of Biology, Faculty of Sciences, Tunis El Manar University, Tunis, Tunisia

## Abstract

Human ageing is a complex trait that involves the synergistic action of numerous biological processes that interact to form a complex network. Here we performed a network analysis to examine the interrelationships between physiological and psychological functions, disease, disability, quality of life, lifestyle and behavioural risk factors for ageing in a cohort of 3,270 subjects aged ≥55 years. We considered associations between numerical and categorical descriptors using effect-size measures for each variable pair and identified clusters of variables from the resulting pairwise effect-size network and minimum spanning tree. We show, by way of a correspondence analysis between the two sets of clusters, that they correspond to coarse-grained and fine-grained structure of the network relationships. The clusters obtained from the minimum spanning tree mapped to various conceptual domains and corresponded to physiological and syndromic states. Hierarchical ordering of these clusters identified six common themes based on interactions with physiological systems and common underlying substrates of age-associated morbidity and disease chronicity, functional disability, and quality of life. These findings provide a starting point for indepth analyses of ageing that incorporate immunologic, metabolomic and proteomic biomarkers, and ultimately offer low-level-based typologies of healthy and unhealthy ageing.

## Introduction

The rate at which the human population is ageing is a global concern. In 2013, the worldwide proportion of elderly people aged ≥60 years was reported as 11.7%, and this proportion is projected to reach 21.1% by 2050^1^. In developed countries in particular, this rise is associated with a fall in the mortality rate, due to factors such as improvements in lifestyle, health-care, and disease management, and a decline in the fertility rate. Concomitant with an ageing population is an increase in the rate of non-communicable and chronic diseases that is characteristic of the functional decline of the human body with increasing age. These combined effects exert economic pressure on society as the demands for healthcare services increase, and places a burden on the relatively shrinking working population.

Ageing is a complex trait that involves the interaction between numerous, fundamental biological processes, such as inflammation, cellular and immune senescence, mitochondrial dysfunction, and reduced stress resistance. The mechanisms that underlie these processes are vast, and although individually have only a modest effect on ageing, they interact in a complex network to support homeostatic and reproductive functions^2^. These mechanisms act across multiple organ systems and underlie an age-associated decline in physiological function and susceptibility to numerous diseases, resulting in functional disabilities, a poor quality of life, and increased mortality. By applying an integrative, systems biology approach, we can better explore how these biological mechanisms contribute to the ageing process at an individual and population level.

Multi-morbidity is the co-occurrence of two or more chronic diseases in an individual that is most common in elderly patients and is a well-recognized example of the complexity of multi-organ system interactions. Notable examples of interactive biological networks that are often implicated in multi-morbidity include the neuro-endocrine and psycho-immunological axes (specifically, the hypothalamo-pituitary axis (HPA) and the sympathetic-adreno-medullary (SAM) axis) that underlie stress, mental disorders, psychosomatic disorders, and frailty^3–6^, and the poorly understood cardio-renal axis that underlies combined heart failure and kidney injury^7^. A better understanding of the clustering of different diseases in an individual would provide insights into common underlying biological mechanisms, which would help guide the design of new therapeutic approaches and improve patient management.

Studies of age-associated multi-morbidity at employ cluster analyses or inquiry of large-volume datasets in health system databases are being performed by a small, but growing number of research groups^8–13^. Most studies have based their analyses on hospital medical diagnoses, which although identify disease clusters, do not include geriatric syndromes or physiological and functional measures of health states, such as body mass, blood pressure, glucose levels, muscle strength or cognitive performance^14^. We propose that a population-based phenotypic network analysis of age-associated health and functional status and disease risk factors might provide a richer insight into the links between physiological and psychological function, disease, disability, and quality of life. A network analysis of health and disease phenotypic connections may also be used in conjunction with molecular and genetic data to unravel the complexity of age-associated health and disease states, thus enabling the identification of disease modules and pathways, and molecular relationships among distinct pathophysiological phenotypes^15^.

In this study we examined the network clustering structure that underlies physiological, psychological, disease, functional, lifestyle, behavioural, and socio-cultural variables that contribute to ageing. These variables were obtained from the ongoing Singapore Longitudinal Ageing Study (SLAS) and were used to construct a network representation of the complex “biopsychosocial” phenotype of health, that considers all relevant determinants of health and disease (as defined by the World Health Organization). Specifically, we examined data from the second cohort of the SLAS^16,17^, SLAS-2, which included 3,270 subjects aged ≥55, who are residents in the south-central and southwest regions of Singapore. The measured variables span across domains, and include, but are not limited to, patient data for physical and cognitive capability, dietary and medical histories, social status, psychological states, and metabolic and immunological information. Our analysis brings together category-valued and numeric-valued variables under a single unified framework, allowing us to obtain both pairwise interrelationships, clustering information and group commonalities between disparate data types coming from a widespread set of domains.

## Full Network-Derived Clusters

**Figure 1:**
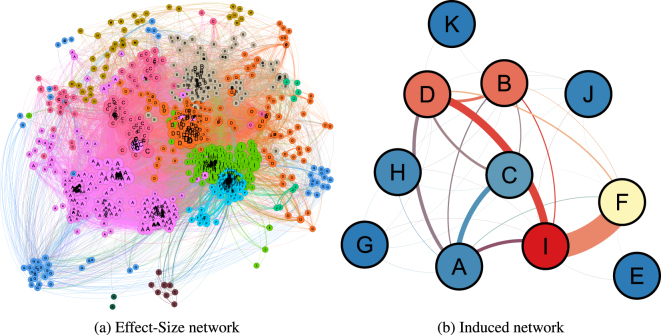
(**a**) A pairwise effect-size network constructed from SLAS-2 data (*N* = 1157, *E* = 62806) and (**b**) its induced network, showing the clustering structure. Nodes represent variables in (**a**), and clusters of variables in (**b**). Node labels give the cluster each variable belongs to in (**a**). The size of each corresponds to its degree (number of neighbouring variables and clusters in (**a**,**b**) respectively), and the colour corresponds to the cluster a variable is assigned to using the Louvain community detection algorithm^18^ in (**a**), and the cluster’s betweenness centrality (BC) in (**b**). Graph visualisations were done using **Gephi** ^47^.

Using the SLAS-2 data, we constructed a pairwise effect-size network (Fig. 1), and obtained 11 clusters of variables using the Louvain communnity detection algorithm^18^. These clusters are mixed-domain clusters, and may correspond to a broad-scope structuring of the data.

We label each cluster manually by examining the contents of each cluster (Extended Data Fig. 1). Of the eleven clusters obtained, Cluster I (Age and Education, Visuo-Spatial-Verbal Learning and Recall) is the most central cluster, with betweenness centrality (BC) equal to 0.13, followed by Clusters B (Metabolic Syndrome, Hypertension, and Inflammation) and D (Demographical, Physical and Nutritional Information), (BC = 0.11). The rest of the clusters, in order of descending BC are:: Cluster F (Dementia and Cognitive Impairment) −0.07; Cluster C (Health Status and Depression) −0.02; Clusters A (Activities of Daily Living, Dementia, and Cognitive Impairment) and H (Pulmonary Dysfunction and Other Medical Conditions) −0.008; Cluster J (Memory Span and Inflammatory Response) −0.004; and Clusters E (Memory Intrusion Errors), G (Supplements, Alternative Medicine, and Tea), and K (Weight Loss), all with BC = 0.

## MST-Derived Clusters

**Figure 2:**
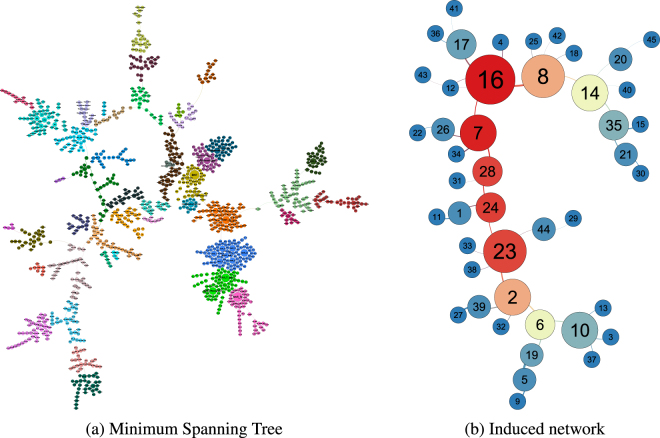
(**a**) Minimum spanning tree (MST) of a pairwise effect-size network constructed from SLAS-2 data (Fig. 1) and (**b**) its induced network, showing the clustering structure. Nodes represent variables in (**a**), and clusters of variables in (**b**). The size of each corresponds to its degree (number of neighbouring variables and clusters in (**a**,**b**) respectively), and the colour corresponds to the cluster a variable is assigned to using the Louvain community detection algorithm^18^ in (**a**), and the cluster’s betweenness centrality (BC) in (**b**). Graph visualisations were done using **Gephi** ^47^.

From a minimum spanning tree (MST) of the effect-size network, we obtained 45 clusters of variables, which are described in more detail below (Fig. 2 and detailed in the Supplementary Information). The clusters we obtained from the MST correspond to the fine-grained clustering structure of the effect-size network, as we show below by a correspondence analysis between the sets of clusters obtained in this section and the previous one.

## Central Clusters

The 45 clusters obtained from the MST could be generally classified as central and non-central clusters, according to their betweenness centrality^19,20^ (BC) in the induced network of the MST (Fig. 2). The BC of a network node is a measure of how how important the node is to the entire network, expressed in terms of how many pairwise shortest-paths it lies across; in a cluster network where the nodes denote clusters, edges denote the presence of association between clusters, and the edge weights denote the “distance” (inversely-proportional to strength of association) between connected clusters, a high-BC node denotes a cluster which is strongly associated to the rest of the clusters, as compared to those clusters in turn. The prominent central clusters (Clusters 16, 7, 24, 28, and 23) in our network lay on a single line of nodes forming the backbone of the cluster network. They were predominantly clusters composed of physiological and syndromic states associated with lipid metabolism, cardio-renal and cardio-pulmonary ageing, frailty, and cognitive impairment (Table S1 and Extended Data Fig. 2). These clusters are described in more detail below.

### Cluster 16: Lipid Metabolism

Cluster 16 is the most central cluster of the network. This cluster contains variables associated with body mass (weight, waist-to-hip ratio, the body mass index (BMI), and the circumferences of the subjects’ middle upper arms, calves, and hips) and diagnoses of central obesity. It also includes lipid concentrations (LDL-cholesterol, HDL-cholesterol, total cholesterol:HDL cholesterol ratio, triglycerides, and diagnoses of dyslipidaemia), subjects’ medical histories regarding high cholesterol (including physician visits and medications), and criterion measures of metabolic syndrome (Table S2). One of the five criterion-based measures of frailty proposed by Fried *et al*.^21^, shrinkness, is contained in this cluster.

### Cluster 7: Nutrition, Cardio-Renal and Cardio-Pulmonary Dysfunction

Cluster 7 contains variables denoting subjects’ demographic information (employment status, and for those retired, reasons for voluntary or involuntary retirement), physical characteristics (gender, height, knee-to-floor height, and supine arm length), nutritional status (folate, vitamin B12 and homocysteine levels), red blood cells (haemoglobin, haematocrit, red blood cell volume and distribution width), and markers of renal dysfunction (eGFR and creatinine). It also contains variables encoding risk factors for chronic obstructive pulmonary disease (smoking and job experience which entailed exposure to dust, fumes, gas, or vapours) and physical activity (duration of daily light and heavy household tasks, and recent walks). Table S3 lists all the variables contained in this cluster.

### Cluster 24: Frailty and Exhaustion

Cluster 24 contains physiological and functional measures of age-associated decline in physical function, namely, the duration of time in arising and fast gait speed tests, as well as self-reported daily times in performing sitting, light and moderate activities, and sleeping durations. Furthermore, it contains subjects’ self-perceptions of tiredness and being worn out. It also contains three out of the five criterion-based measures of physical frailty (slowness, exhaustion, and physical inactivity) proposed by Fried *et al*. (Table S4).

### Cluster 28: Physical Strength

Cluster 28 contains measures of physical strength (hand grip strengh and knee extension). It also contains the fifth criterion-based measure proposed by Fried *et al*. (weakness), as a bridge node connecting the rest of the cluster with Cluster 24, which as mentioned above contains three other criterion-based measures (Table S5).

### Cluster 23: Cognitive Impairment

Cluster 23 contains variables pertaining to memory and cognitive function: the Mini Mental-State Examination (MMSE) and the backward digit-span test. Also included are several variables encoding the subjects’ demographic information, such as age and educational attainment. A group of variables in the cluster pertain to the use of computers and the Internet, in particular their use in playing mind and brain stimulation games. Finally, the cluster contains measures of visual acuity, in close association with the subjects’ histories of having eye problems (Table S6).

## Non-Central and Peripheral Clusters

A total of 25 peripheral clusters, so-called based on their location to the outermost edges of the induced tree network, had a BC equal to zero (Fig. 2, Table S7). All but three of these clusters can be considered single-domain clusters; the exceptions are Clusters 3 (Depression, Dementia and Neurodegenerative Disorders), 4 (Osteoporosis, Stroke, Diabetes, Gastrointestinal Conditions and Alternative Medicine), and 42 (Diabetes, Hypercholesterolaemia, and Hypertension).

Peripheral clusters form part of the group of non-central clusters in the cluster tree network. Aside from them, there are ten other non-central clusters in this group, with BC values ranging from 0.05 (Clusters 1, 5, 20, 21, 26, 39, 44) to 0.41 (Clusters 2 and 8, which flank the line of central clusters along the tree network’s spine).

### Neurocognitive and Neurodegenerative Domains

Fourteen clusters share neurocognitive and neurodegenerative domain variables. Twelve of these are anchored by Cluster 2 (Visual and Motor Recall) to the central Cluster 23 (Cognitive Impairment). Taken together, they constitute a major branch of the cluster network. In Cluster 2, scores for the Boston Naming Test (BNT) and the Colour Trails Test (CTT), with their corresponding *Z*-scores, are present.

Clusters 27 and 39, a pair of linked clusters, branch off Cluster 2. Cluster 39 connects to Clusters 2 and 27, while Cluster 27, a peripheral cluster, is connected to Cluster 39 alone. Both deal with subjects’ verbal learning and recall, and comprise result variables from the Story Recall Test (SRT). Cluster 32 (Visuo-Spatial Processing) contains those for the Clock Reading Test (CRT), and is another peripheral cluster branching off Cluster 2. The remaining eight form two branches and are anchored by Cluster 6 (Visuo-Spatial Memory) to Cluster 2. This cluster contains scores for the revised Brief Visuospatial Memory Test (BVMT-R).

Clusters 5 (Auditory Learning and Recall I), 9 (Auditory Learning and Recall II) and 19 (Auditory Learning and Recall III) are connected in a line configuration which branches off Cluster 6. All three contain variables from the Rey Auditory Verbal Learning Text (RAVLT). Cluster 19, connected to Cluster 2, contains the scores for the five trials administered as well as the total score. Cluster 5, connected to it and Cluster 9, contains repetition errors. The peripheral Cluster 9 contains intrusion errors and false-positive scores. RAVLT intrusion errors are known to be associated with ageing-related neurodegenerative diseases and dementia^22^, while false-positive errors may underlie mechanisms reflecting episodic memory process and disinhibition deficits in subjects with Alzheimer disease and behavioural variant fronto-temporal dementia in an Indian-Australian cohort study, but was insufficient in distinguishing between the two conditions^23^.

Cluster 10 (Dementia), together with three peripheral clusters connected to it (3, 13 and 37), form the other branch connected to Cluster 6. Cluster 10 contains evaluations of dementia and cognitive decline and impairment in subjects, both self-reported (via the MMSE) and third-person informants (the Informant Questionnaire on Cognitive Decline in the Elderly, IQCODE). Cluster 3 (Neurodegenerative Disorders) brings together assessments of dementia, Alzheimer disease, Parkinson’s disease and other neurodegenerative disorders. Cluster 13 (Depression) is indicative of subjects’ depression and state of life satisfaction. Cluster 37 (Memory Decline) is associated predominantly with subjective memory and cognitive complaints.

The thirteenth, Cluster 38 (Memory Span), contains scores for the forward digit span test. It is connected to the central Cluster 23 (Cognitive Impairment), which contains the corresponding scores for the backward digit span test. This cluster may be an artifact cluster; in the MST of the variables it connects directly to the section of Cluster 23 containing the backward span trial scores, with an effect-size (Spearman’s *r* ^2^) weight of 0.09.

The last one, Cluster 1 (Lack of Vitality), is connected to another central cluster, Cluster 24 (Frailty and Exhaustion). The two share variables associated with lack of energy and vitality, with Cluster 1’s contents leaning towards self-reports and self-perceptions, and those of Cluster 24 leaning more into physical measurements, as discussed above. Also included within the cluster are subjects’ responses to the GDS and SF-12 surveys regarding self-perceptions of energy and vitality, as well as their total GDS score. Despite belonging to the neurocognitive domain, it is connected to Cluster 11 (Physical Health Status).

### Health Status and Quality of Life Domains

This group comprises clusters that are mostly on the other end of the cluster tree backbone as those of the neurocognitive and neurodegenerative domains anchored by Cluster 23. Many of these clusters are mixed-domain clusters.

Clusters 4, 12, and 43 are peripheral clusters branching off Cluster 16 (Lipid metabolism). Cluster 4 (Osteoporosis, Stroke, Diabetes, and Gastrointestinal Conditions) for example, denote an individual’s medical history across several conditions such as diabetes mellitus, osteoporosis, and stroke, and can be characterised as pertaining to the disability status of the patient. Cluster 12 (Inflammation) groups together primary markers of inflammation, including C-reactive protein (CRP), interleukin-6 (IL-6) and tumour necrosis factor (TNF). Also included in the cluster are counts of white blood cells associated with inflamation: basophils, eosinophils, lymphocytes, monocytes, neutrophils and total white blood cell count, and subjects’ histories of undergoing surgery, with the procedures cited predominantly associated with inflammation (appendectomy, Caesarian section, and hysterectomy). Cluster 43 (Weight Loss) denote unexpected weight loss in subjects over varying periods of time.

A fourth cluster branches off Cluster 16: Cluster 17 (Diabetes and Chronic Obstructive Pulmonary Disease). This cluster contains medical records and diagnoses pertaining to diabetes and chronic obstructive pulmonary disease (COPD), two conditions which have been found to be in close association, suggesting similar patho-physiological processes^24^. Branching off this cluster, in turn, are two peripheral clusters: Cluster 41 (Chronic Obstructive Pulmonary Disease) which is composed of reports of symptoms of COPD such as extended-term cough and phlegm, and Cluster 36 (Medical Conditions), containing subjects’ medical records concerning asthma, cancer, hypertension and hypercholesterolaemia.

From Cluster 16, the cluster network’s backbone extends along Clusters 8 (Chronic Multi-Morbidity and Hypertension) and 14 (Activities of Daily Living, Eye, and Respiratory Conditions), with their own branching non-central and peripheral clusters. Cluster 8 is composed of variables giving the number of co-morbid conditions in the subjects, as well as their frailty indices measured using a cumulative deficit approach^25,26^. It also contains subjects’ diagnoses of hypertension through measurements of blood pressure, medical history and intake of anti-hypertensive medicines. Attached to it are three peripheral clusters of closely-associated variables: Cluster 18 (Medical Prescription Sites) which contains the locations from which the subjects obtained their prescription medicines (family clinic, hospital specialist, or polyclinic) for four medical conditions they specify (of which hypertension, hypercholesterolaemia, diabetes mellitus and cardiac conditions are the most frequently-cited). Cluster 25 (Chronic Heart Disease) brings together variables pertaining to heart disease, atrial fibrillation and heart failure (medical history, physician visits, hospitalization, and diagnosis) and those pertaining to thyroid problems. Cluster 42 (Diabetes, Hypercholesterolaemia, and Hypertension), similarly, groups together information on the duration subjects have taken medicines for the four medical conditions they specified above, as well as medical histories of hypertension and hypercholesterolaemia.

Cluster 14 (Activities of Daily Living, Eye, and Respiratory Conditions) has a strong physical function component. It groups together variables pertaining to subjects’ capabilities in performing basic daily activities (such as getting up from bed, washing, dressing, and going outdoors) with their medical histories of debilitating physical conditions such as arthritis, gout, and backache. The inclusion of variables associated with eye problems such as cataract and respiratory infections may indicate that they are contributing factors to impaired physical function. Continuing the trend of ADL-oriented clusters, Cluster 35 (Activities of Daily Living Disabilities) and its attached clusters, 15 (Activities of Daily Living, Tiredness) and 21 (Activities of Daily Living) bring together variables pertaining to an individual’s ADL disabilities. Cluster 30 (Physical Mobility) is a peripheral cluster connected to Cluster 21 and contains assessments of an individual’s balance and gait ability using the Performance-Oriented Mobility Assessment (POMA) tool.

Cluster 31 (Physical Endurance) contains information regarding subjects’ physical endurance, in the form of the average distance walked and number of steps climbed on weekdays and weekends. It is separated from the clusters above, and is instead strongly-connected to the central Cluster 28 (Physical Strength).

Other clusters belonging to these domains, but branch off Cluster 16, include Clusters 20 (Supplements) and 45 (Medication Adherence), branching off from Cluster 14, and Clusters 29 (Tea Consumption Habits), 33 (Social Support Network), and 44 (Curry and Tea Consumption Habits), which branch off Cluster 23.

## Cluster Correspondence

In addition to the clusters obtained from the minimum spanning tree, we applied modularity-based clustering to the full network (*N* = 1157, *E* = 62806) and obtain 11 clusters, as shown in Fig. 1. The clusters obtained from the full network are mixed-domain clusters, except for Clusters E (a subset of the MST-derived Cluster 9) and K (which is identical to Cluster 43). The relationship between the clustering labels obtained from the full network and those from the MST is moderate, even accounting for chance (AMI = 0.46).

We use the Jaccard distance, obtained from the Jaccard similarity index^27^ to identify which of the 11 clusters correspond the most to a given MST-derived cluster. Since the sizes of the full-network clusters are in general larger than the MST-derived clusters, we expect that several of the latter would be subsets of the former. To check this, we use the *subset similarity index*, which is the Jaccard similarity index normalised by its expected value if the two sets examined form a subset-superset pair. Of the 45 MST-derived clusters, 22 are subsets of their corresponding full-network cluster (Table S8). MST Cluster 43 (Weight Loss) is identical to the corresponding full-network Cluster K, while Cluster 9 (Auditory Learning and Recall II) is a superset of the full-network Cluster E. Furthermore, the relationship of another 8 MST clusters to their corresponding full-network clusters are at least 90% (but below 100%) similar to a subset-superset relationship, and for another 4 MST clusters, between 80% and 90%. Thus, four-fifths of the MST clusters (36 of 45) are subsets, or very close to it, of their corresponding full-network clusters. This indicates that the MST, by keeping the strongest associations or effect sizes, may lend itself to a more fine-grained clustering result compared to its parent network.

## Edge Groups

We next examined the clusters, represented by the nodes at the end of each edge in the induced network tree, in order to test whether they have domain and thematic commonalities. We identified the common thematic groups, classified the edges according to each group, and grouped the cluster-nodes according to whether they shared edges of a given group. Consequently, the resulting grouped cluster-nodes shared common themes.

**Figure 3:**
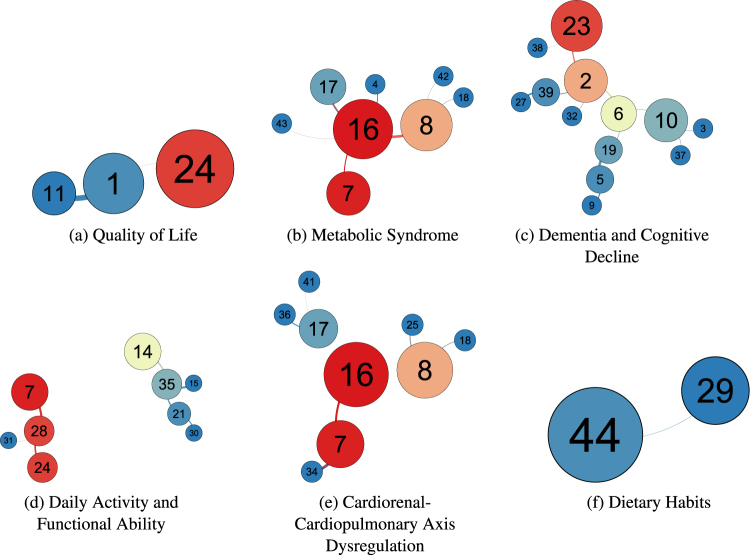
The six cluster edge groups comprising the cluster tree network, along with the commonalities of each edge group. The size of each node corresponds to its degree (number of neighboring clusters), and the colour of each node corresponds to its betweenness centrality (BC). Edge group labels were made by manually-inspecting the content variables of each cluster.

To this effect, we identified six common-themed edge groups in our analysis of the MST and the induced tree network, and grouped the nodes accordingly (Fig. 3). These edge groups do not have mutually-exclusive membership, as several edges can belong to more than one group, meaning that the cluster-nodes linked by these edges may share more than one commonality.

The common themes represented by the 6 cluster-edge groups may be meaningfully labelled in terms of axes of multi-organ physiological system interaction and common underlying substrates for age-related morbidity and disease chronicity, functional disability and quality of life. Two groups (Metabolic Syndrome and Cardiorenal and Cardiopulmonary Axis Dysregulation) are of most interest due to their centrality in the entire network.

Not all associations within a cluster and with adjoining clusters are necessarily informative or lend themselves to straightforward interpretation. Some observed associations with some disease or syndrome clusters, and especially with lifestyle and behaviour, such as the use of nutritional supplements and complementary and alternative medicines are difficult to interpret or even spurious. As the direction and nature of influence or causality between any associative pair is not explicit, these statistical associations do not imply causality.

## Discussion

In this network analysis we found that the complex biopsychosocial phenotype of health and disease in old age may be understood as a hierarchical network of inter-relationships between physiological and psychological functions, disease, disability, quality of life, and lifestyle and behavioural risk factors. Our findings imply that the most important physiological endophenotypes for health in ageing lie along the backbone of a branching network of relationships with other central and peripheral phenotypes, with extracellular transport, glucose, lipid, and caloric metabolism, and osmotic and acid-base balance being the most important in particular (Cluster 16: *Lipid Metabolism* and Cluster 7: *Nutrition*, *Cardio*-*Renal and Cardio*-*Pulmonary Dysfunction*), and the other backbone clusters, concerned with ageing-associated frailty, exhaustion, and cognitive deficits, playing a subsidiary, but still somewhat central, role (Cluster 28: *Physical Strength*, Cluster 24: *Frailty and Exhaustion*, and Cluster 23: *Cognitive Impairment*). This backbone branches out to other physical, cognitive, and psychosocial phenotypic manifestations of health and disease. A number of known disease or syndromic clusters, such as metabolic syndrome, cardio-renal syndrome, dementia, and neurodegenerative diseases are recapitulated in these cluster networks.

An example of known and reported associations can be found in Cluster 17 (Diabetes and Chronic Obstructive Pulmonary Disease). The extra-pulmonary complications of COPD are now well accepted, and it is considered a risk factor for new-onset type 2 diabetes mellitus through a series of patho-physiological mechanisms, including inflammation and oxidative stress, and the mechanisms involved in the development of both may be similar^24,28–32^.

In conclusion, the biopsychosocial phenotype of health and disease in old age may be described in terms of the complex inter-relationships between physiological and psychological functions, disease, disability, quality of life, and lifestyle and behavioural risk factors within an organized hierarchical cluster network of relationships. Central to these relationships are physiological and syndromic states (namely frailty, and metabolic, cardio-renal, cardio-pulmonary, and neurocognitive disorders), which could be meaningfully cross-mapped into common themes. These themes represent axes of multi-organ, physiological system interactions and underlying substrates of age-associated morbidity and disease chronicity, functional disability, and quality of life.

## Methods

**Figure 4:**
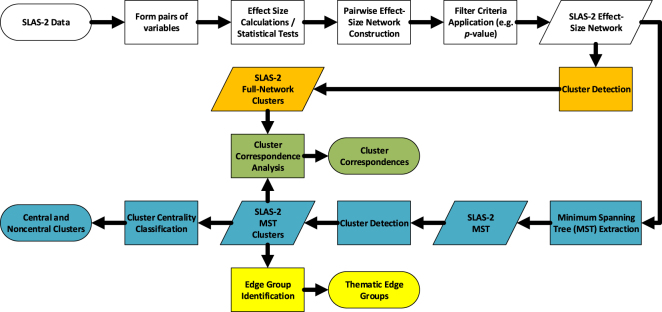
Workflow of procedures undertaken in the present study. Colours represent sections of the methodology and analyis. Ovals represent input and output data; rhomboids, intermediate data representations; rectangles, methods and analyses.

In this section we describe the methods and measures used in the present study. The series of steps performed in the analysis, including input, intermediate and output data, is depicted in Fig. 4.

### Study Participants and Measurements

The study participants form the second cohort of the ongoing Singapore Longitudinal Ageing Studies (SLAS), here termed SLAS-2. This study measured the characteristics of 3,270 residents of south-central and south-west Singapore, aged 55 years and above. The characteristics measured for this study are spread across the psychosocial, lifestyle and behavioural, biomedical and physical, and neurocognitive domains, along with assessment metrics for dementia. The SLAS study, and its methods, have been described previously^16,17^; a brief description is presented here.

Study participants were identified using a door-to-door census, and participated voluntarily in the study after invitation. The response rate for the invitation was 78.5%. Participating subjects completed an extensive range of baseline interviews, standard tests for cognitive function, physical examinations, and analyses of blood samples. The interviews spanned a range of domains, including (but not limited to) demographic information (such as age, gender, ethnicity, educational level, and employment status), nutritional intake and dietary habits (kinds of food consumed, tea drinking habits), medical histories (including physician visits, hospitalisation, and medicines taken for a list of medical conditions), and quality-of-life, life satisfaction and depression surveys. Tests for cognitive function and memory include the Boston Naming Test (BNT), the revised Brief Visuospatial Memory Test (BVMT-R), the Mini Mental State Examination (MMSE), the Colour Trails Test (CTT), the Rey Auditory Verbal Learning Test (RAVLT), and the Story Recall Test (SRT), among others. Physical examinations and measurements include measuring hand grip strength, knee extension tests, balance and gait tests (the Performance-Oriented Mobility Assessment, POMA), and standard body measurements (such as height, weight, body mass index, blood pressure, and waist and thigh circumferences). Finally, measurements from blood samples taken from subjects include those of red and white blood cells, albumin, creatinine, eGFR and several markers for inflammation (c-reactive protein, interleukin-6, and TNF-*α*).

Approval for this study was granted by the Institutional Review Board of the National University of Singapore, and all study participants provided informed consent in writing prior to obtaining their data. Furthermore, all methods have been performed in accordance with all relevant rules and regulations.

### Pairwise Effect-Size Calculations

The analysed data set consisted of 1,581 variables (683 numerical and 898 categorical). A complete data set was not available for all participants included in SLAS-2, which is discussed in more detail below.

We used a minimum spanning tree (MST) network derived from a pairwise effect-size distance representation of the data and decomposed it into maximum-modularity clusters. Our approach is broadly similar to the weighted correlation network analysis methods developed by Hovarth *et al*.^33–39^, but instead, we determined the threshold for the network edges using statistical significance. We considered the categorical and numerical variables simultaneously by using the effect size, which was calculated from the respective statistical tests instead of the correlation coefficient that applies only to pairs of numerical variables. In cases of a pair of numerical variables there was the further advantage of the effect size being the square of the correlation coefficient (*r* ^2^).

We classified the variables as either numerical or categorical, and formed all possible variable pairs, whilst removing for each pair the subjects with missing and/or invalid data. We then performed a correlation analysis for each pair to determine the variables that were significantly correlated. We used non-parametric versions of the standard correlation measures and significance tests (Spearman rank and Kruskal-Wallis analysis of variance, respectively), in order to minimize assumptions regarding the underlying data. The exact statistical correlation measure and significance test depended on the composition of the pair (Table 1). *Z*_*max*_ is the maximum of the *Z*-scores obtained in Dunn *post hoc* test and *n* is the number of samples used for the Kruskal-Wallis analysis of variance. We choose these effect-size measures due to their property of being constrained between 0 and 1. As Cramer’s *ϕ* exhibits a large bias for finite samples, we use the correction proposed by Bergsma^40^.

**Table 1:** Statistical measures of correlation used in the analysis, according to the different types of variables in each pair.

Pair Composition	Statistical Test	Effect Size Measure
Both numerical	Student’s *t* test on Spearman’s *r*	Spearman’s *r* ^2^
Both categorical	*χ* ^2^ test on contingency table	Cramer’s *ϕ* ^2^
One numerical, one categorical	Kruskal-Wallis ANOVA + Dunn’s *post hoc* test	${Z}_{max}^{2}/n$

A sample-size calculation for correlations to obtain the minimum needed to detect a small effect size (Cohen’s *d* equal to 0.1) at 80% statistical power yielded 790 subjects. We discarded any tests that were invalidated due to an insufficient number of subjects, or any variable in the pair having the same value for the subjects remaining, after removing those with missing or invalid entries. We then performed significance testing on the calculated pairwise effect-size measures. The statistical test used on a pairwise effect-size depended on the type of variables comprising the pair (Table 1). A significance level threshold *α* = 0.05 was chosen and a Bonferroni correction was made for multiple comparisons. This threshold was divided by 1,248,990 (the number of unique pairs among the 1,581 variables), resulting in an effective *α* of 4.01 × 10^−8^. A computed effect-size measure was considered significant if the *p*-value did not exceed this modified threshold.

### Network Clustering and Analysis

From the pairwise statistical tests we were left with 62,806 statistically-significant effect-size calculations which satisfy the minimum sample-size criterion for our 1,581 variables. We then constructed a network using the variables as the nodes. These nodes were linked by an edge if a correlation between them was statistically-significant. In the latter case, the weight we chose for the edge was either the effect-size measure itself, or a distance transformation of the effect-size measure: if *R* ^2^ denotes the effect-size measure, the distance weight we assign is then (1 − *R* ^2^)/*R* ^2^. Following this calculation, the distance between any two variables with a high calculated effect size is low, and vice versa. We termed this the “effect-size distance” between the two variables. Our choice of using either the effect-size or the distance transformation as the weight depended on the further procedures we applied to the network, as discussed below.

A total of 1,157 variables (589 categorical, 568 numerical) in the constructed network belonged to a “giant component”, where any two variable nodes were linked by at least one path. The remaining variables are single, or isolated variables with no significant correlation to any other variable after the Bonferroni correction was applied.

This study only utilised the giant component that was generated from our analyses (Fig. 1), and from it we constructed a minimum spanning tree (MST) using Kruskal’s algorithm^41^. The MST derived from a parent network contains all the nodes belonging to its parent, and all its edges are likewise taken from the latter. Such an MST, therefore, contains information on the hierarchical clustering of the underlying network^42^. We are interested in the tree which contains the strongest effect sizes, and therefore associations, in the parent network. In such a structure, the nodes corresponding to the variables can be clustered in a hierarchical manner, with nodes positioned close to each other (variables that share a higher pairwise effect-size) being grouped together. To achieve this, instead of using the effect-size *R* ^2^ values as edge weight inputs to Kruskal’s algorithm, we use the distance transformation previously mentioned, (1 − *R* ^2^)/*R* ^2^. This way, the algorithm, which iterates through the network edges in order of increasing value of the supplied edge weights, captures the “minimum-distance”, and therefore maximum effect-sizes, first. We use the algorithmic implementation which is part of the **NetworkX** library for the Python programming language^43^.

We next applied the Louvain community detection algorithm^18^ on both the giant-component network and the MST in order to cluster the variables in each network further. This algorithm identifies the partitioning of a network that corresponds to a maximum of the modularity measure. If we consider a network and a partitioning of the nodes into putative clusters, the edges of the network will connect the nodes inside a putative cluster (known as intra-cluster edges) or nodes to different clusters (known as inter-cluster edges). The modularity of the network partitioning is then the degree to which the intracluster and intercluster edges are denser or sparser, respectively, than would be the case if the edges were randomly-distributed among the nodes in the network. The modularity also measures how strongly the nodes inside each potential cluster are linked as compared to nodes in different clusters. The modularity has a value between −0.5 and 1.0, with 0 corresponding to the null scenario of a random distribution of edges. This time, instead of using the distance transformation of *R* ^2^ as the input edge weight for the algorithm, we use *R* ^2^ itself, since it is indicative of pairwise closeness.

From the full giant component network, we obtain 11 clusters with a moderately-high modularity of 0.54. The clusters obtained from the full network are mostly mixed-domain clusters. From the MST, we obtained 40 clusters with a very high modularity measure of 0.93, which reflects the high degree of clustering that is characteristic of a MST. Clusters obtained from the MST are generally smaller than those obtained from the full network.

### Cluster Correspondence Analysis

To establish the relationship between the clusters obtained from the two networks (full network and MST), we perform a correspondence analysis between the two sets of clusters. Our correspondence analysis makes use of the concept of *Jaccard similarity* ^27^, which is a measure of overlap between a pair of sets. Given two finite, nonempty sets **X** and **Y**, Equation 1 gives the Jaccard similarity between the two sets:

1 $$J({\bf{X}},{\bf{Y}})=\frac{|{\bf{X}}\cap {\bf{Y}}|}{|{\bf{X}}\cup {\bf{Y}}|}$$

where |**X**| and |**Y**| are the cardinalities of **X** and **Y**, respectively. *J* can have values between 0 and 1; in practice its upper limit depends on the relative sizes of **X** and **Y**:

2 $${J}_{max}({\bf{X}},{\bf{Y}})=\frac{\min \,\{|{\bf{X}}|,|{\bf{Y}}|\}}{\max \,\{|{\bf{X}}|,|{\bf{Y}}|\}}$$

*J*_*max*_ is obtained when either **X** or **Y** is the subset of the other, and equals unity when the two sets are identical.

The Jaccard distance, *D*_*J*_(**X**, **Y**) = 1 − *J*(**X**, **Y**) is a metric over the collection of finite, nonempty sets^44,45^. Thus, we can use it to compare the closeness of pairs of clusters, and determine which full-network cluster an MST-derived cluster is closest to.

A second measure, the *subset similarity index*, *j*(**X**, **Y**) = *J*(**X**, **Y**)/*J*_*max*_(**X**, **Y**) describes how close **X** and **Y** are to being a subset-superset pair. We find that nearly half of the MST-derived clusters (22 out of 45) are strict subsets of corresponding full network-derived ones. The rest overlap in some way with a full-network cluster. Of these, in one case the MST cluster is identical to one of the full-network clusters, and in the other the MST-derived one is the *superset* of a corresponding full-network cluster.

Finally, to determine the strength of relationship between the clustering of the full network and that produced of the MST (which share identical nodes by definition), we calculate the *adjusted mutual information* (AMI) between the cluster labels produced from the two networks. The AMI ranges from zero (indicating that any association between the two sets of labels is purely due to random assignment) and unity (indicating that the two sets of labels are equivalent). We use the AMI implementation of the **scikit**-**learn** Python library^46^.

### Cluster Centrality Classification

Finally, we calculated the betweenness centrality (BC) for each node in the induced network. The BC^19,20^ of a node in a network is a measure that serves to rank its position in the network, and is a measure of its importance. BC is calculated using the following formula, where *i* is the node under consideration, *j* and *k* are other nodes in the network, *σ*_*jk*_ is the number of shortest paths in the network from *j* to *k*, and *σ*_*jk*_(*i*) is the number of shortest paths from *j* to *k* which pass through *i*:

3 $$BC(i)=\sum _{j\ne i\ne k}\,\frac{{\sigma }_{jk}(i)}{{\sigma }_{jk}}$$

A node with a high BC in a network sits on a higher proportion of shortest-paths between any two other nodes than a node with a low BC. In our induced network, this means that the variables comprising a node (cluster) with high BC have strong effect sizes (either directly or indirectly) with more of the remaining variables in the larger, spanning tree network, as the paths are guaranteed by the MST to be the shortest effect-size distance paths, and thus have a correspondingly strong pairwise effect size. The BC of a cluster-node in the induced network is a measure of its importance in comparison with the other cluster-nodes.

We classify the cluster in accordance with the betweenness centrality of their corresponding nodes in the induced (cluster tree) network. We use two classes of clusters: central clusters and noncentral clusters, with peripheral clusters as a subset of the latter class. An examination of the BC values of the cluster-nodes yields 0.5 as a natural cutoff between the two classes; in our work the BC is normalised such that it takes a value between 0 and 1, and a gap in the BC of about 0.12 is present between the two clusters on either side of the cutoff (0.53 and 0.41 for Clusters 23 and 8 respectively). We define peripheral clusters as the noncentral clusters with a BC of zero.

### Edge Group Identification

We examined the variables that comprised each MST cluster, as well as variables in adjoining clusters, to determine if they shared any common points. In addition, by representing each cluster as a node, we could extract the network consisting of the identified clusters as nodes and the connections between the bridge nodes from adjacent clusters as edges (the “induced network”). The induced network served as a schematic representation of the clusters identified in the MST, and additionally allowed us to analyse the relative importance of each cluster. Due to being applied to a tree-structured network, the network we obtained was also a tree structure, where the nodes represent the clusters and the edges represent the inter-cluster links found in the parent MST, weighted according to a combination of the inter-cluster edges present. Only an edge connects two adjacent clusters in the parent tree, therefore, the edge weight in the induced network is given by that of the corresponding edge in the parent. We were then able to examine the contents of each cluster as well as the edges between clusters to identify common links.

## Electronic supplementary material

Supplementary Information
