# Supplementary material for: Health and disease phenotyping in old age using a cluster network analysis
Source: Sci Rep. 2017 Nov 15;7:15608. doi: 10.1038/s41598-017-15753-3 (PMC5688160; doi:10.1038/s41598-017-15753-3)
Supplement: Supplementary file 1 — Supplementary Information [file 41598_2017_15753_MOESM1_ESM.pdf]

Health and disease phenotyping in old age using a cluster network analysis

Jesus Felix Valenzuela[1], Christopher Monterola[1], Victor Joo Chuan Tong[2], Tze Pin Ng[3*] and Anis Larbi[4,5,6,7,8*+]

[1]*Institute of High Performance Computing, Computing Science Department, 1 Fusionopolis Way, #16-16 Connexis North, 138632, Singapore*

[2]*Institute of High Performance Computing, Social and Cognitive Computing Department, 1 Fusionopolis Way, #16-16 Connexis North, 138632, Singapore*

[3]*Yong Loo Lin School of Medicine, National University of Singapore, Department of Psychological Medicine, 1E Kent Ridge Road, NUHS Tower Block, Level 9, 119228, Singapore*

[4]*Singapore Immunology Network, 8A Biomedical Grove, Immunos Level 4, 138648, Singapore*

[5]*Department of Medicine, University of Sherbrooke, Quebec, Canada*

[6]*Yong Loo Lin School of Medicine, National University of Singapore, Department of Microbiology and Immunology, Singapore*

[7]*School of Biological Sciences, Nanyang Technological University (NTU), Singapore*

[8]*Department of Biology, Faculty of Sciences, Tunis El Manar University, Tunisia*

[*]*Equal senior authors for this work.*

[+]*To whom correspondence should be addressed: anis_larbi@immunol.a-star.edu.sg*

Supplementary Information

This section contains a description of each variable for the 45 MST-derived clusters that were identified in this analysis. We have categorized the clusters into central and non-central clusters (of which peripheral clusters are a special case) according to their betweenness centrality (BC). Clusters with BC above 0.50 are classified as central clusters, while those with BC below 0.50 are classified as non-central clusters, and those with BC equal to zero are designated peripheral clusters. For the non-central clusters, we grouped them according to which central cluster they branch off from. The most significant characteristic of the contained variables is provided for each cluster in the form of its cluster name or title together with the cluster connections. Extended Data Figures 1 and 2 are schematic diagrams of how the full network and MST-derived clusters, respectively, are connected together.

Central Clusters

Five central clusters were identified in this analysis. These clusters and their cluster connections are detailed in Table S1.

Table S1: Central clusters. *All five clusters lie on the backbone of the structure of the derived cluster network, with Cluster 16 being the most central. Abbreviation: BC, betweenness centrality.*

Cluster	Name	Cluster Connections	BC
**16**	*Lipid Metabolism*	4, 7, 8, 12, 17, 43	0.58
**7**	*Nutrition, Cardio-Renal, and Cardio-Pulmonary Dysfunction*	16, 26, 34	0.57
**24**	*Frailty and Exhaustion*	1, 23, 28	0.54
**28**	*Physical Strength*	7, 24, 31	0.53
**23**	*Cognitive Impairment*	2, 24, 33, 38, 44	0.53

The key, characteristic variables for each of the central clusters are indicated in Tables S2-S6.

Table S2: Variables of Cluster 16 – Lipid Metabolism. *Abbreviations: BMI, body mass index; HDL, high density lipoprotein; LDL, low density lipoprotein; IDF, International Diabetes Federation; NCEP, National Cholesterol Education Program.*

**Variable Group**	**Variables**
**Body mass**	BMI; Middle upper arm, hip, calf circumference; Waist-to-hip ratio. Unintended weight loss of 4.5 kg in last 6 months.
**Lipids**	LDL-Cholesterol; HDL-Cholesterol; Total Cholesterol:HDL Cholesterol,ratio; Dyslipidaemia; Triglycerides; Use of lipid-lowering medications.
**Metabolic syndrome**	Central obesity; Raised triglyceride levels; Reduced HDL levels. Metabolic syndrome diagnoses (IDF and NCEP)
**Clinical status**	High cholesterol (medical history, physician visits, medication treatment)
**Frailty component**	Shrinkness.
**Depression**	Diagnosis of depression.

Table S3: Variables of Cluster 7 – Nutrition, Cardio-Renal, and Cardio-Pulmonary Dysfunction. *Abbreviation: COPD, chronic obstructive pulmonary disease.*

**Variable Group**	**Variables**
**Nutrients**	Folate, Vitamin B12.
**Haematological (iron) status**	Haemoglobin; Haematocrit; Mean corpuscular haemoglobin concentration; Mean corpuscular volume; Red blood cell concentration and distribution width.
**Cardio-renal status**	Creatinine; eGFR; Homocysteine; Chronic kidney disease determination.
**COPD risk factors**	Employment at jobs with high exposure to dust, fumes, gas, or vapours; smoking
**Employment status**	Employment status; Working status; Reason for involuntary/voluntary retirement.
**Physical markers**	Gender; Height (total and knee-to-floor); Supine total arm length.
**Physical activity**	Daily duration of performing light/heavy household tasks; Times of walk in past 2 weeks; Duration of recent walks.
**Eye conditions**	Detached retina or eye surgery in past 1 month.
**Coffee**	Frequency of coffee drinking.

Table S4: Variables of Cluster 24 – Frailty and Exhaustion.

Variable Group	Variables
**Frailty criteria**	Slowness; Exhaustion; Low level of physical activities; Total score; Categorization (healthy, pre-frail, frail).
**Balance and gait**	Time to arise; Time taken for Fast Gait Speed Test.
**Physical activity (weekday and weekend)**	Sitting activity; Light activity; Moderate activity. Days in week performing light household tasks.
**Sleep**	Duration of sleep and lying down (weekdays and weekends); Average duration of night sleep; Number of needed hours of sleep.
**Quality of life**	Feeling tired; Feeling worn out.

Table S5: Variables of Cluster 28 – Physical Strength.

Variable Group	Variables
**Hand grip strength**	Left hand, trials 1 - 3; Right hand, trials 1 - 3
**Knee extension**	Left knee, trials 1 - 3; Right knee, trials 1 - 3

Table S6: Variables of Cluster 23 – Cognitive Impairment. *Abbreviation: MMSE, Mini Mental State Examination.*

Variable Group	Variables
**Age and education**	Age; Years of schooling; Completed education level; Age-educational level composite grouping
**Visual acuity**	Logmar chart, left and right eyes; Medical history of eye problems.
**Computer and internet use**	Use of computers, video games, personal digital assistants (PDA), mind or brain stimulation games.
**MMSE**	Total score; Multiple MMSE item variables.
**Leisure-time activities**	Computer games; Any cognitively-stimulating activities.
**Digit span test**	Backward span trials 1 and 2 subtotal scores; Backward trials Z-score.
**Cognitive impairment**	Memory decline: reading for over 5 minutes at a time; Baseline cognitive impairment determination.

Table S7: Non-central and peripheral clusters. *Abbreviations: ADL, Activities of Daily Living; COPD, Chronic Obstructive Pulmonary Disease.*

Cluster	Variables Contained	Cluster BC	Neighboring Clusters
**Cluster 8: Chronic Multi-Morbidity and Hypertension**	32	0.4123	14, 16, 18, 25, 42
**Cluster 2: Visual and Motor Recall**	42	0.4112	6, 23, 32, 39
**Cluster 14: ADL, Eye and Respiratory Conditions**	33	0.2886	8, 20, 35, 40
**Cluster 6: Visuo-Spatial Memory**	65	0.2865	2, 10, 19
**Cluster 10: Dementia**	42	0.1332	3, 6, 13, 37
**Cluster 35: ADL Disabillities**	31	0.1321	14, 15, 21
**Cluster 17: Diabetes and COPD**	28	0.0899	16, 36, 41
**Cluster 1: Lack of Vitality**	23	0.0888	11, 24
**Cluster 19: Auditory Learning and Recall II**	90	0.0888	5, 6
**Cluster 5: Auditory Learning and Recall I**	66	0.0455	9, 19
**Cluster 20: Supplements**	24	0.0455	14, 45
**Cluster 21: Activities of Daily Living**	23	0.0455	30, 35
**Cluster 26: Leisure-Time Activities and Dietary Habits**	50	0.0455	7, 22

Table S7: Non-central and peripheral clusters. *Abbreviations: ADL, Activities of Daily Living; COPD, Chronic Obstructive Pulmonary Disease.*

Cluster	Variables Contained	Cluster BC	Neighboring Clusters
**Cluster 39: Verbal Learning and Recall II**	32	0.0455	2, 27
**Cluster 44: Curry and Tea Consumption Habits**	6	0.0455	23, 29
**Cluster 3: Neurodegenerative Disorders**	28	0	10
**Cluster 4: Osteoporosis, Stroke, Diabetes, and Gastrointestinal Conditions**	20	0	16
**Cluster 9: Auditory Learning and Recall Errors**	44	0	5
**Cluster 11: Physical Health Status**	24	0	1
**Cluster 12: Inflammation**	21	0	16
**Cluster 13: Depression**	28	0	10
**Cluster 15: Activities of Daily Living, Tiredness**	38	0	35
**Cluster 18: Medical Prescription Sites**	4	0	8
**Cluster 22: Sleep**	12	0	26
**Cluster 25: Chronic Heart Disease**	16	0	8

Table S7: Non-central and peripheral clusters. *Abbreviations: ADL, Activities of Daily Living; COPD, Chronic Obstructive Pulmonary Disease.*

Cluster	Variables Contained	Cluster BC	Neighboring Clusters
**Cluster 27: Verbal Learning and Recall I**	34	0	39
**Cluster 29: Tea Consumption Habits**	14	0	44
**Cluster 30: Physical Mobility**	31	0	21
**Cluster 31: Physical Endurance**	4	0	28
**Cluster 32: Visuo-Spatial Processing**	14	0	2
**Cluster 33: Social Support Network**	16	0	23
**Cluster 34: Pulmonary Function**	15	0	7
**Cluster 36: Medical Conditions**	16	0	17
**Cluster 37: Memory Decline**	12	0	10
**Cluster 38: Memory Span**	4	0	23
**Cluster 40: Alcohol Consumption**	8	0	14
**Cluster 41: Chronic Obstructive Pulmonary Disease**	7	0	17
**Cluster 42: Diabetes, Hypercholesterolaemia, and Hypertension**	6	0	8
**Cluster 43: Weight Loss**	3	0	16

Table S7: Non-central and peripheral clusters. *Abbreviations: ADL, Activities of Daily Living; COPD, Chronic Obstructive Pulmonary Disease.*

Cluster	Variables Contained	Cluster BC	Neighboring Clusters
**Cluster 45: Medication Adherence**	4	0	20

Table S8: Correspondences between clusters obtained from the full network ($N = 1157, E = 62806$) and its minimum spanning tree (MST), with the Jaccard distance and the subset similarity index between each cluster pair. *MST-derived clusters are smaller than their corresponding full-network clusters, excepting Cluster 9 (the superset of Cluster E, marked with* **)*

Full Network Cluster	MST Cluster	Jaccard Distance	Subset Similarity
A	10	0.8306	0.9722
	15	0.8471	0.9697
	21	0.9046	1.0000
	30	0.8852	0.8921
	35	0.8760	0.9637
	37	0.9502	1.0000
B	8	0.7581	0.9224
	16	0.7642	0.9588
	17	0.8806	0.5203
	18	0.9672	1.0000
	25	0.9134	0.6604
	42	0.9508	1.0000
C	1	0.8205	0.8974
	3	0.8468	0.6293
	11	0.8120	0.9010
	13	0.7565	1.0000
	22	0.8957	1.0000
D	7	0.7987	0.8970
	24	0.8882	0.7583
	26	0.7439	0.7990
	28	0.9167	1.0000

Table S8: Correspondences between clusters obtained from the full network ($N = 1157, E = 62806$) and its minimum spanning tree (MST), with the Jaccard distance and the subset similarity index between each cluster pair. *MST-derived clusters are smaller than their corresponding full-network clusters, excepting Cluster 9 (the superset of Cluster E, marked with *)*

Full Network Cluster	MST Cluster	Jaccard Distance	Subset Similarity
D	31	0.9744	1.0000
	33	0.9317	0.6661
	34	0.9038	1.0000
	40	0.9487	1.0000
	44	0.9615	1.0000
E	9*	0.8182	1.0000
F	5	0.6580	1.0000
	19	0.5337	1.0000
G	4	0.8676	0.3772
	20	0.6034	0.9418
	29	0.7544	1.0000
	45	0.9298	1.0000
H	14	0.8939	0.1286
	36	0.6000	1.0000
	41	0.8250	1.0000
I	2	0.8037	1.0000
	6	0.7143	0.9407
	23	0.8936	0.4949
	27	0.8411	1.0000
	32	0.9346	1.0000
	39	0.8505	1.0000

Table S8: Correspondences between clusters obtained from the full network ($N = 1157, E = 62806$) and its minimum spanning tree (MST), with the Jaccard distance and the subset similarity index between each cluster pair. *MST-derived clusters are smaller than their corresponding full-network clusters, excepting Cluster 9 (the superset of Cluster E, marked with *)*

Full Network Cluster	MST Cluster	Jaccard Distance	Subset Similarity
J	12	0.8400	0.4200
	38	0.5000	1.0000
K	43	0.0000	1.0000

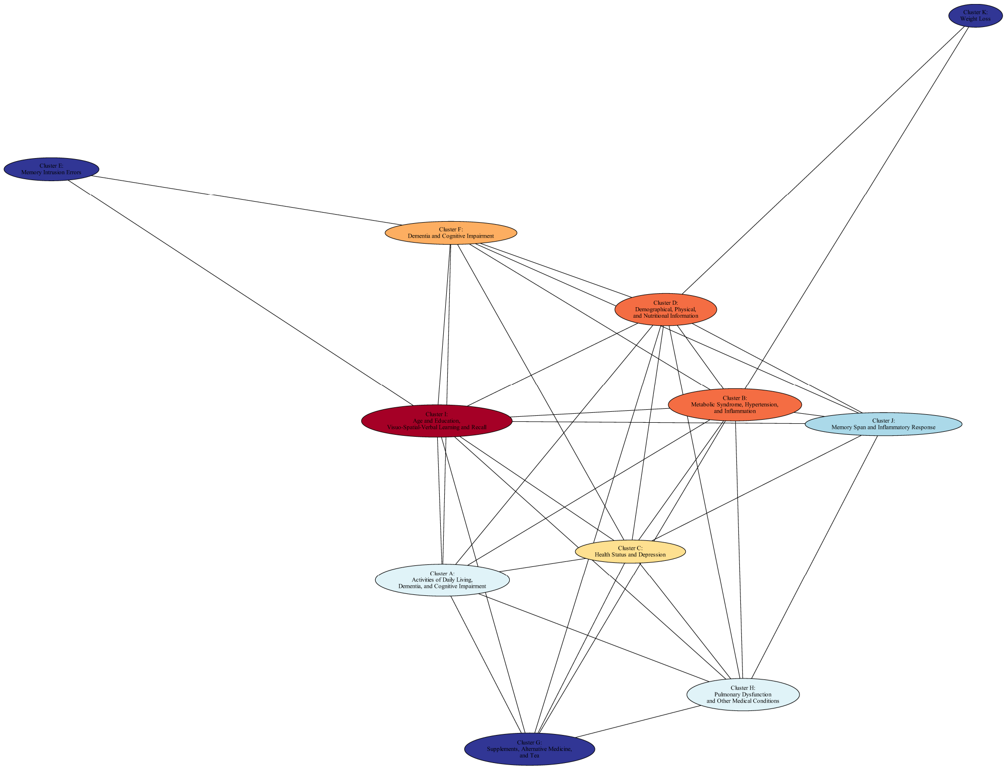

Extended Data Figure 1: Schematic diagram of clusters obtained from a pairwise effect-size network constructed from SLAS-2 data in Figure 1 ($N = 1157, E = 62806$)**.** Cluster labels were manually-curated through examination of the variables contained by each. Node colours represent relative levels of the clusters' betweenness centrality (BC), from high BC (dark red) to low BC (low BC).

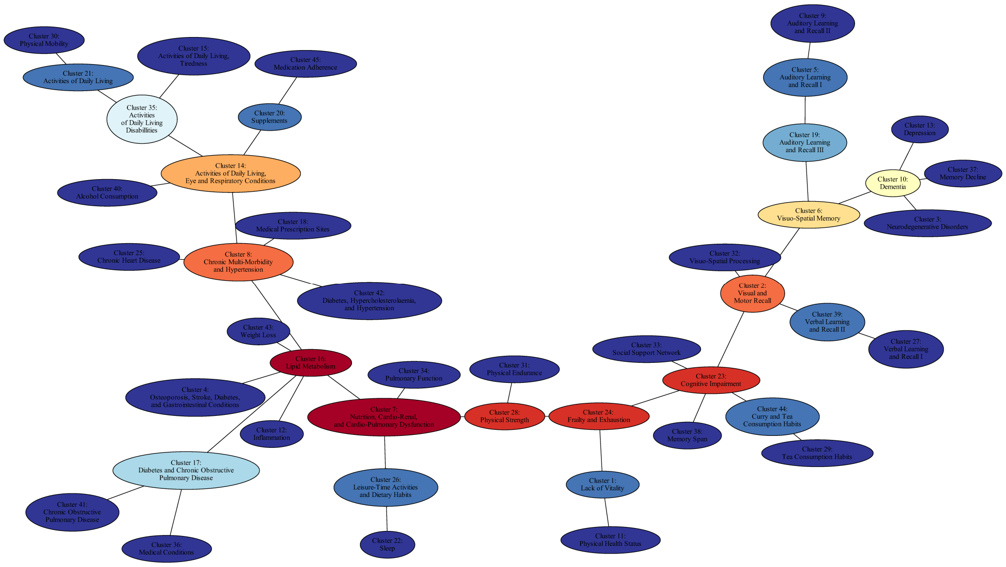

Extended Data Figure 2: Schematic diagram of clusters obtained from the minimum spanning tree of the effect-size network. Cluster labels were manually-curated through examination of the variables contained by each. Node colours represent relative levels of the clusters' betweenness centrality (BC), from high BC (dark red) to low BC (low BC).
